# The Technological Process of Obtaining New Linen Dressings Did Not Cause the Loss of Their Wound-Healing Properties

**DOI:** 10.3390/ma14247736

**Published:** 2021-12-15

**Authors:** Tomasz Gębarowski, Izabela Jęśkowiak, Maciej Janeczek, Magdalena Żuk, Agnieszka Dobosz, Benita Wiatrak

**Affiliations:** 1 Department of Medical Science Foundation, Wroclaw Medical University, Borowska 211, 50-560 Wroclaw, Poland; tomasz.gebarowski@upwr.edu.pl (T.G.); agnieszka.dobosz@umw.edu.pl (A.D.); benita.wiatrak@umw.edu.pl (B.W.); 2 Department of Biostructure and Animal Physiology, Wroclaw University of Environmental and Life Sciences, Kożuchowska 1/3, 51-631 Wroclaw, Poland; maciej.janeczek@upwr.edu.pl; 3 Department of Pharmacology, Wroclaw Medical University, Mikulicza-Radeckiego 2, 50-345 Wroclaw, Poland; 4 Department of Genetic Biochemistry, University of Wroclaw, Przybyszewskiego 63/77, 51-148 Wroclaw, Poland; magdalena.zuk@uwr.edu.pl

**Keywords:** linen dressings, flax fabrics, healing wound

## Abstract

Background: Linen dressings were invented a few years ago but are still being worked on. Methods: The obtained fabrics from the traditional variety of flax (Nike), two transgenic types of flax (M50 and B14) and the combination of these two flax fibers (M50 + B14) were tested in direct contact in cell cultures. Cell viability tests were performed, and the proliferation potential of cells on Balb3T3 and NHEK cell lines was checked using the Sulforhodamine-B (SRB) test. Moreover, the effect of new linen fabrics on apoptosis of THP-1 cells, as well as on the cell cycle of NHEK, HMCEV and THP-1, cells after 24 h of incubation was assessed. Results: All tested linen fabrics did not raise the number of necrotic cells. The tested fabrics caused a statistically significant decrease in the total protein content in skin cancer (except for 0.5 cm of Nike-type fabrics). The smallest cells in the apoptotic phase were in cultures treated with M50 fiber on an area of 0.5 cm. After 48 h of incubation of HEMVEC, NHEK and THP-1 cells with the tested fabrics, the growth of S-phase cells was noticed in all cases. At the same time, the greatest increase was observed with the use of B14 fabric. Necrosis is not statistically significant. Conclusions: All the obtained flax fibers in the form of flax dressings did not lose their wound-healing properties under the influence of the technological process. New dressings made of genetically modified flax are a chance to increase the effectiveness of treatment of difficult healing wounds.

## 1. Introduction

The huge variety of wounds and the wide range of dressings available make it difficult to choose the right wound treatment. The most difficult problems occur in the treatment of chronic wounds, which include thermal and chemical burns, frostbite, wounds caused by carcinoma, hematological, immune wounds, and pyoderma, as well as venous ulcers, ischemic wounds, diabetic foot syndrome and bedsores. These are wounds that heal for more than 8 weeks. In the local treatment of wounds, hydrocolloid, alginate, hydrogel, dextranomer dressings, polyurethane foam, semi-permeable dressings and dextranomer with a polyurethane membrane, as well as linen dressings, are used [1].

Problems with wound healing are related with a variety of diseases, such as diabetes, atherosclerosis, and immune disorders. Chronic wounds are a serious medical issue, and because of that, researchers conducted several preclinical studies on a flax-fiber wound dressing. This material was selected in part because of its high hygroscopic, anti-inflammatory properties and high antioxidant content that promotes wound healing. Cannabidiol CBD (synergy with β-sitosterol) is responsible for wound-healing properties, while the effect on matrix remodeling activity depends mainly on the phytosterol content [2]. β-sitosterol promotes migration to human endothelial cells and has a pro-angiogenic effect [3] and induces the proliferation of keratinocyte cell lines [4]. Both sterols and terpenophenol—cannabidiol (CBD) are responsible for the anti-inflammatory effect of flax fiber [2]. CBD inhibits the expression of IL1, IL6, MCP-1, TNFα and NFκB [5]. Additionally, the CBD reduces the propagation of inflammation by the inhibition of COX 2 and NOS expression [6,7].

The earliest information on the medical use of linen dressings comes from Mesopotamia [8]. Among the many descriptions of the use of flax, the modification of the dressing with the use of honey is particularly interesting [9]. The use of linen bandages to support fresh meat applied to the wound is also interesting [10]. The Ebers papyrus and Edwin-Smith papyrus show that linen bandages were used to treat wounds and fractures [11]. The Ebers papyrus also mentions the use of linen sutures to close wounds [12]. Linen was also known in Greco-Roman medicine. Hippocrates recommended the use of flax fiber to stabilize loose teeth and as a dressing for various types of wounds [13]. Thus, flax is a plant whose medical use has many centuries of tradition. This was because of its properties, which, when used skillfully, brought good results.

Work on linen dressings was started by Polish scientists. The using genetic bioengineering methods, they developed a completely new type of flax dressings enriched with antioxidants. The basis for the linen dressings described in this publication is several types of genetically modified flax. All these types are examples of fiber flax (starting variety Nike) and are a source of biologically active fiber.

Type M flax plants overproduce polyhydroxybutyrate (PHB). A safe material for medicine originates from transgenic flax plants, which produce both components of the flax/PHB composites [14,15]. Conversely, transgenic plant type expressing beta-1,3-glucanase (one of the PR2 genes) has improved the plant’s defense against pathogens, which makes it easier to grow this type of plant [16].

The combination of the excellent test results of both types of transgenic plants appeared to be the way to obtain super-plants. Since carrying out multiple transformations is usually very difficult (especially since a complicated three-gene construct was used to generate M plants), it was decided to carry out this experiment by crossing methods. In order to produce a new type of flax, which would be characterized by the synthesis of a polymeric macromolecular compound, polyhydroxybutyrate (PHB), thanks to which the obtained fiber should have improved biomechanical and biochemical parameters, as well as having properties derived from B type plants with increased expression of the β-glucanase gene, that is, enhanced anti-pathogenic resistance and increased accumulation of polyamines.

In this study, fabrics from the traditional variety of flax (Nike) and two transgenic types of flax (M50 and B14) and combinations of these two flax fibers (M50 + B14) were obtained and then tested in direct contact with cell cultures. The aim of the research was also to assess whether the technological process of obtaining the linen fabrics did not cause the loss of the healing properties of the flax fibers.

## 2. Materials and Methods

### 2.1. Plants

The procedure for the generation and selection of transgenic flax type M has been described previously. For the genetic transformation of flax (cv. Nike), a construct containing three bacterial genes (*Ralstonia eutropha*) necessary for polyhydroxybutyrate (PHB) synthesis, was used together with a promotor specific to vascular boundless and plastidial targeting sequence [16].

Transgenic plant type B(14) was generated as described previously [10]. The flax (cv. Nike) plants were transformed using constructs bearing potato (*Solanum tuberosum*) gene encoding beta-1,3-glucanase under the control of 35SCaMV promotor.

Generation of MB plants by the simple crossing method, where M was the maternal form, and B was paternal. Flowers of maternal and paternal forms that were in the appropriate stage of ontogenetic development were selected for crossing.

The obtained plants were amplified to obtain the appropriate amount of material for analysis and obtain oil and flax straw.

The unmodified (Nike), transgenic plants (M50 and B14) and MB plants grown in the field were harvested after 4 months. After that, the seeds and straw were collected.

#### 2.1.1. Preparation of Oil from Seeds

Flaxseed oil (5 kg) was cold pressed using an industrial screw oil press (Oil Press DD85G–IBG Monoforts Oekotec GmbH & Co., Berlin, Germany). The obtained oil was stored (until use) at a temperature of 4 °C, and the oxygen supply was limited with gaseous ni-trogen.

#### 2.1.2. Preparation of Fiber from Field Grown Plants (Stems)

The stems that remained after ungrating were loosely decomposed in the open field. The straw was turned every 2 weeks. During this time, bacteria and fungi (which had free access to the plant tissue) degraded the polysaccharides of the cell wall and the middle lamella, thus releasing the flax fibers.

### 2.2. Common Fabric—Technological Process

Flax fiber (derived from M50, B14, MB plants respectively) was rinsed using warm (50–65 °C) demineralized water and subjected to continuous twisting and stretching treatment in order to obtain yarn and then fabric. The whole process, called wet spinning, takes 20 min to 2 h depending on the quality (thickness, elasticity, homogeneity) of the flax fiber. Moist (loosened) flax fiber is more susceptible to machining, so the described procedure (standardly used in the weaving industry) allows to obtain homogeneous yarn.

Before using the thus obtained fabric for biomedical purposes (or research in cell culture), a sterilization step is necessary.

### 2.3. Tested Linen Fabric

**Figure 1:**
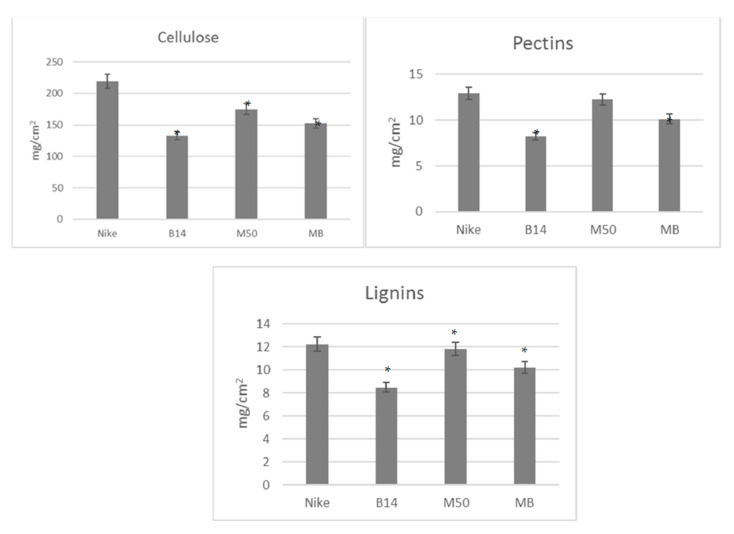
The content of the main polymers in the studied fabrics. The statistical significance of the differences between the results for the tested linen fabrics compared to the control was calculated using the Tukey post hoc test (* *p* < 0.05).

The obtained fabric of the traditional variety of flax (Nike) and two transgenic types of flax (M50 and B14) and the combination of these two flax fibers (M50 + B14) were tested in direct contact (Figure 1) in cell cultures. The fabrics with an area of 1 cm were placed in the medium above the cell culture. The fabrics prepared in this way had the following grammages: respectively 23 mg, 20.5 mg, 14.6 mg and 17.5 mg Nike, M50, B14 and the combination of M50 with B14. The fabric was sterilized for 1 h by UV irradiation and then added to the culture for 48 h.

### 2.4. Determination of Polimers Content

The cellulose content was determined using the colorimetric method with the reagent anthrone, as described [17]. The total lignin content was determined by the acetyl bromide method [18]. Determination of the pectin and hemicellulose content was performed using the acetyl bromide method [17,18].

### 2.5. Biochemical Analysis of Fabric

#### 2.5.1. Phenolic Compound Extraction

Ten grams of linen fabric was used for phenolic compound extraction, as described in [15].

#### 2.5.2. UPLC-PDA-MS Analysis of Phenolic Components

Phenolic compounds were measured using the Waters Acquity UPLC System with a 2996 PDA detector and Waters Xevo Qtof MS System mass spectrometer, as described previously [19].

#### 2.5.3. Hydrophobic Compounds Extraction and Analysis

Linen fabric (15 g) was extracted with chloroform (3 × 50 mL), and the supernatants after centrifugation (5000 rpm, 10 min) were combined and dried in a pure nitrogen atmosphere. The extract was filtered through a 0.25 μm Acrodisc. The analysis was performed with a Waters Acquity Ultra Performance Liquid Chromatograph with a PDA and mass detector as described previously [2].

### 2.6. Antioxidant Capacity Determination

#### 2.6.1. DPPH Scavenging Assay

The methanol extract (obtained from 1 g of fabric) was thoroughly mixed with 0.1 mM DPPH and then incubated at 37 °C for 30 min. The absorbance of the solution was measured at 515 nm.

#### 2.6.2. TBARS Assay

For analysis, 1 g of the previously grounded in Retsch MM200 milling fabric was collected and extracted three times with 1 mL of methanol in an ultrasonic bath for 20 min. The extract was centrifuged at 18,000 rpm for 10 min at room temperature. The combined supernatants were vacuum dried and re-suspended in 1 mL of methanol. The level of TBARS (thiobarbituric acid-reactive substances) was measured according to the published protocol [20].

### 2.7. Cell Line and Conditions

Cell lines were purchased from Lonza (Basel, Switzerland): Human Normal Dermal Fibroblast (NHDF), Human Dermal Microvascular Endothelial Cells—Adult (HMVEC) Cell. BALB/3T3 mouse fibroblasts (clone A31 from mouse embryo donor) were from Sigma-Aldrich. Normal human epidermal keratinocytes (NHEK) were obtained from PromoCell. Epidermal carcinoma and monocyte cell line (THP-1) were obtained from ATCC (Manassas, VA, USA). A431 cells were derived from epidermoid carcinoma and purchased from EATCC. All cells were grown at 37 °C, in 5% CO_2_, 95% humidity with morphology evaluation. If confluence exceeds 70%, cell cultures were passaged using the TrypLe solution, counted using a Brucker chamber and seeded in 96-well plates for assay. If the confluence was less than 70%, the medium was replaced with fresh. NHDF were cultured in Dulbecco’s modified Eagle medium (DMEM) without phenol red, NHEK were grown in the KBM-Gold medium (Keratinocyte Cell Basal Medium). A431 were incubated in Dulbecco’s modified Eagle’s medium (DMEM). BALB/3T3 were cultured in Minimum Essential Medium (MEM). THP-1 cells were incubated in an RPMI-1640 medium. HMVEC were grown in an EGM medium, which was enriched according to Lonza’s procedure. Other media were supplemented with 10% fetal bovine serum (FBS), penicillin (10,000 U/mL), streptomycin (10 mg/mL), and L-glutamine (200 mM).

### 2.8. Cell Viability

After direct contact of the linen fabric with Balb/3T3 cells for 24 h, the viability of the cell culture was assessed. After removing the fabric, the supernatants from the wells were collected in the previously described tubes. Next, the cells were washed with PBS, which was collected in appropriate tubes. The TrypLE solution was then added to the culture and left at 37 °C for 2 min. The solution was again collected in centrifuge tubes and spun at 600× *g* for 5 min. Then the pellet was resuspended in PBS with propidium iodide for 5 min at RT in the dark. The samples were then transferred to chips and analyzed on an Arthur image cytometer (NanoEnTek Inc., Seoul, Korea). Arthur’s cytometer counts all cells, not just necrotic cells.

### 2.9. Cell Proliferation

The cell proliferation potential was evaluated in the NHDF, NHEK cell lines using the Sulforhodamine-B (SRB) test. After 48 h of incubation with test fabrics, cell cultures were fixed with cold trichloroacetic acid (TCA) for the adherent cell line at 50% concentration and 20% for cells growing in suspension at 4–8 °C for 30 min. The plates were then washed five times under running water, after drying, added sulforhodamine-B dye again for 30 min, and unbound dye was removed for five washes with 1% acetic acid and dried again. Finally, the protein was dissolved in Trisma solution, and the absorbance at 555 nm was measured with a microplate reader (Victor2, PerkinElmer, Waltham, MA, USA). As a control, TCA-fixed co-cell cultures before the use of linen fabrics were used.

### 2.10. Apoptotic Cells

The influence of linen fabric on the apoptosis of THP-1 cells was tested. After a 48 h treatment with the tested fabrics, the fabrics were taken out, washed in PBS, which, together with the supernatant, were transferred into previously called tubes. The culture was also swimming with the PBS that was harvested, and TrypLE solution was used and incubated at 37 °C for 2 min. The separated cell suspension was then collected in tubes and centrifuged at 600× *g* for 5 min. Then the pellets were resuspended in 100 µL HEPES-NaOH buffer, pH 7.5. Then Annexin V-FITC fluorochromes were added and incubated in the dark for 10 min. Cells were transferred to chips and analyzed on an Arthur image cytometer (NanoEnTek Inc.).

### 2.11. Cell Cycle

The cell cycle of NHEK, HMCEV and THP-1 after 24 h treatment with flax fabrics was assessed. After incubating the cells with the test fabric, the cells were detached and centrifuged, and then the pellet was fixed using cold ethanol (70%) for 10 min at room temperature and centrifuged again at 600× *g* for 5 min. The cell pellet was resuspended in propidium iodide solution and again left in the dark for 10 min. Samples were transferred into chips and analyzed on an Arthur image-based cytometer (NanoEnTek Inc., Seoul, Korea).

### 2.12. Statistical Analysis

All biological assays were performed in five independent replications. In addition, one-way ANOVA and Tukey post hoc analyzes were performed. The point of significance was established * *p* < 0.05. Statistical analyzes were performed with Statistica v.13 software.

## 3. Results

### 3.1. The Content of Polymers in the Fabric

We examined the surfaces of the material–cell interaction, not the mass of the dressing. We checked the contact surface of the dressing to the wound. The analyzed fabrics do not show significant differences in relation to the individual main polymers (cellulose, lignin and pectin). The differences in absolute values shown in the graphs (Figure 1) result from the different densities of the analyzed fabrics.

The values are given per fabric surface (1 cm). The statistical significance of the differences between the results for the tested linen fabrics compared to the control was calculated using the Tukey post hoc test (* *p* < 0.05).

### 3.2. Fabric Biochemical Analysis

In order to identify potential differences in the content of bioactive compounds between the previously described fibres [11] and the fabric, a thorough analysis of the biochemical composition of the fabric was carried out, with particular emphasis on compounds showing anti-inflammatory, antioxidant or pro-proliferative effects.

In the process of preparing the fabric for biomedical purposes, the chemical treatment of the yarn (bleaching, leaching, etc.) was given up. However, even with a “gentle process”, some kind of loss is unavoidable by washing out the unstably bound phenolic compounds—this mainly concerns phenolic acids (ferulic acid, coumaric acid, and other low-molecular-weight phenolic compounds), for which we lose about 10–15% of the original amount. Therefore, it seems reasonable to carry out analyzes on the fabric obtained in this way in order to verify its biomedical properties. The treatment of fibers in order to obtain a fabric also affects their hydrophilicity, which may not be without significance for the effects of their operation in the inflammatory environment/wounds/in contact with body fluids.

Three groups of compounds have been considered useful for medical purposes. The first group consists of phenylpropanoids and isoprenoids (as well as antioxidative and anti-inflammatory compounds) with the primary function as free radical scavengers [12] Polyunsaturated fatty acids were also analyzed as metabolites with proven regenerating effects on cell membranes. The third group of compounds that promote cell proliferation are hydrophobic compounds such as cannabidiol, sterols, and 3-hydroxybutyrate, which is an activator of genes critical for the modification of histone and nonhistone proteins [1].

The first group of compounds is represented by phenolic acids, flavonoids and benzoic derivatives [12].

All of the analyzed fabrics are characterized by the presence of phenolic acids (ferulic acid, coumaric acid, or 4-hydroxybenzoic acid)—the lowest content is shown by a fabric based on the B14 range. An important compound with potential anti-bacterial and pro-proliferative activity is vanillin—the lowest amounts (of 17–35% lower than in the other analyzed fabrics) were found in the B14 fabric (Table 1). 

Flavonoids (such as luteolin) may have a conspicuous role in skin disorders prevention because of their antioxidant and anti-inflammatory effects. The M50 fabric has, by far, the highest content of flavons, but slightly less of these compounds were identified in Nike and MB fabrics.

The hydrophobic compounds existing in flax fabrics, and in particular, cannabidiol (present in all analyzed fabrics in the amount from 0.017 to 0.029 µg/cm^2^) is a well-known anti-inflammatory agent [2].

**Figure 2:**
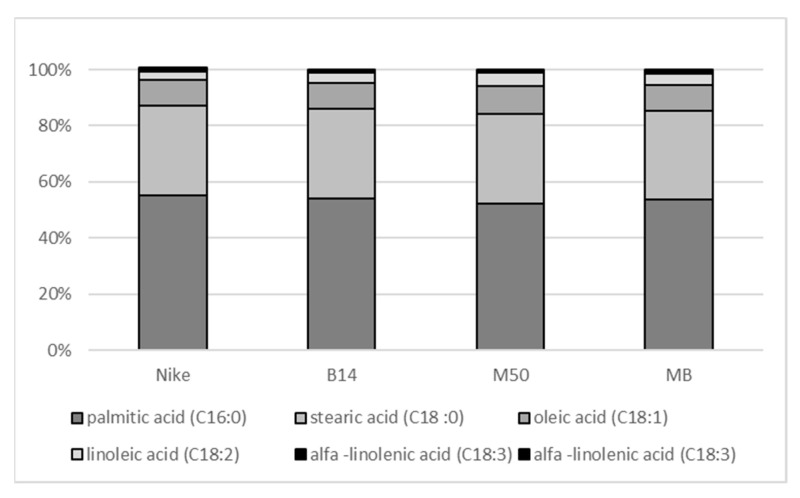
The percentage of fatty acid in the fabric.

The antioxidant properties of the fabrics were tested using two DPPH and FRAP tests (both performed on fabric extracts). The increase in such properties in fabrics derived from modified plants, observed in the DPPH test, most likely results from the presence of phenolic antioxidants. However, the differences in the measured antioxidant activity are not statistically significant. The percentage of fatty acids in the fabric is presented in Figure 2. Only small, statistically insignificant differences in the percentage of individual fatty acids were observed. On the other hand, differences in the absolute content of the individual fatty acids (and thus in their sum) were found (Table 2).

### 3.3. Mechanical Properties of Fabrics and Density

An issue that may have potential significance when using fabric for medicinal purposes is the way the fabric is weaved. It affects not only the biological properties, such as the bioavailability of bioactive compounds, which entails a specific therapeutic effect, but also the mechanical properties of the fabric under consideration.

At the initial stage of planning the experiment, a number of different combinations were prepared, and the mechanical parameters of the obtained fabrics were analyzed. The fabric made of B14-type fiber was characterized by the greatest stiffness.

The physical and mechanical parameters of the obtained fabrics were also analyzed. These properties are the resultant of the characteristics of the fiber used, the process of obtaining yarn and producing (weaving) the fabric. An important parameter for the properties of fabrics (especially for biomedical applications) is the density of the fabric; for the tested fabrics, it oscillated between 0.0238 g/cm^2^ for a fabric made of B14 fibers to 0.0384 g/cm^2^ and 0.0314 g/cm^2^ for a Nike and M50 fabric (respectively). As predicted, the density of the MB fabric was the resultant of the density of the M and B fabrics and equaled 0.0289 g/cm^2^.

The strength of the fabric is also important for medical usability (bandages, compresses, dressings). Stiffness characterizes the resistance of a given material to elastic deformation. Two such parameters were determined for the described fabrics—bending stiffness and tensile stiffness. The fabric B (28 Gpa) turned out to be the most stretchy and the least stretchable one of the M50 and MB yarns—34.9 Gpa. The bending resistance (stiffness) was slightly different: the most flexible was the MB fabric (0.482 Gpa) and the least of Nike (0.787 Gpa).

### 3.4. Cell Viability

**Figure 3:**
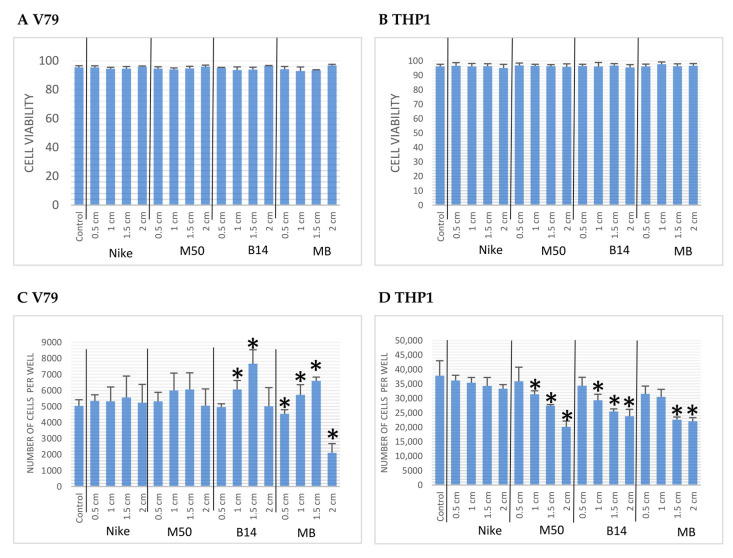
The Balb/3T3 cells viability after 48 h of incubation with the tested linen fabric on four different surfaces (0.5, 1.0, 1.5 and 2.0 cm). The statistical significance of the differences between the results for the tested linen fabric compared to the control was calculated using Tukey’s post hoc test (* *p* < 0.05).

The cytotoxic effect of the tested flax tissues was carried out using the NIH-recommended Balb/3T3 cell line and the monocyte line (THP-1), assessing the number of necrotic cells after staining with propidium iodide. There was no increase in the number of necrotic cells after the treatment of the cell cultures with the tested linen fabrics compared to the control cultures; their number ranges from 4–6% (Figure 3). A lower number of necrotic cells was observed for 2 cm bonded fabrics (MB). In the remaining tested cases, the number of necrotic cells did not exceed 2% (Figure 3A). The greatest number of cells was found in the cultures exposed to 1.5 cm of the surface of the tested fabrics, first B14 fabrics and combinations of M50 and B14, respectively (Figure 3B).

### 3.5. Cell Proliferation

**Figure 4:**
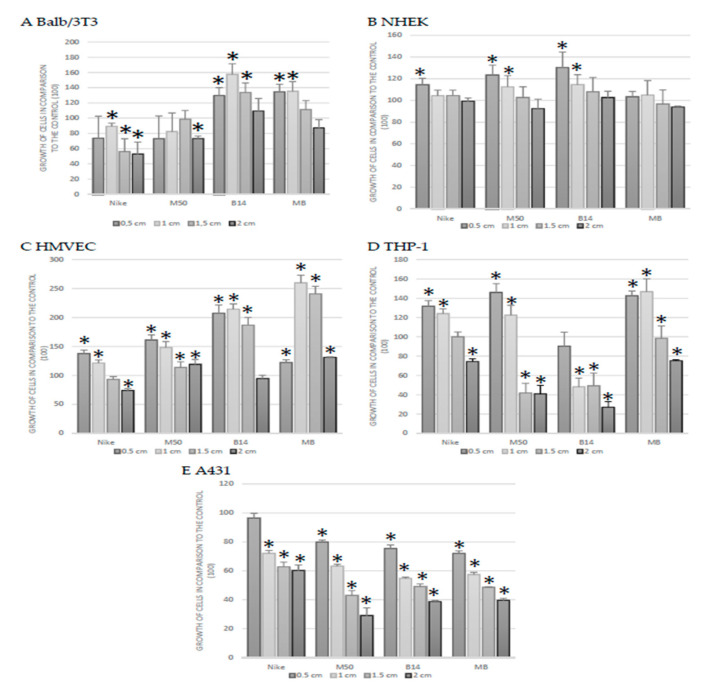
The proliferation of cells (**A**) Balb/3T3, (**B**) NHEK, (**C**) HMVEC, (**D**) THP-1, (**E**) A431 after 48 h of incubation with the tested linen fabrics on four different surfaces (0.5, 1.0, 1.5 and 2.0 cm). The results are the means of 5 independent experiments ± SEM. Statistical significance of differences between the results for the tested linen fabrics compared to the control was calculated using Tukey’s post hoc test (* *p* < 0.05).

The incubation of Balb/3T3 cultures with genetically modified linen fabric and their combination resulted in an increase in the total cell protein in cultures of murine fibroblasts (Balb/3T3) after incubation (48 h) in the entire range of the B14 fabric surface area (except for the largest area of 2.0 cm) and in the range of 0.5–1.0 cm of a combination of genetically modified fabrics M50 with B14. In Balb/3T3 cell culture, a reduction in total protein was observed after incubation with areas of 1.0–2.0 cm of Nike fabric and 2.0 cm of M50 fabric (Figure 4A). Significant reductions in protein levels were found in HMVEC cells, only in the largest area of 2.0 cm of Nike fabric (Figure 4B). At a surface area of 0.5–1.5 cm, an increase in total cellular protein was observed for all flax fibers compared to the control (except 1.5 cm Nike fabric). In addition, the area of 2.0 cm of the M50 fabric and its combination with B14 caused a statistically significant increase in the amount of total protein of HMVEC cells. In the area of 0.5–1.0 cm, the total NHEK cell protein increase was significant after incubation of the culture with M50 and B14 fibers and only 0.5 cm of Nike fabric (Figure 4C). In the remaining surfaces and fabrics tested, no reduction in total protein below the control was observed. The number of monocytes was reduced in all tested fabrics on the largest tested surfaces (1.5–2.0 cm) of the material.

On the other hand, smaller areas increased the total protein of monocytes after using Nike fabrics, M50 and the combination of M50 with B14. The B14 fabric reduces the amount of total protein across the entire range of surfaces tested (Figure 4D). The tested fabrics caused a statistically significant decrease in total protein in skin cancer (except for 0.5 cm of Nike-type fabrics) (Figure 4E).

### 3.6. Apoptosis Cells

**Figure 5:**
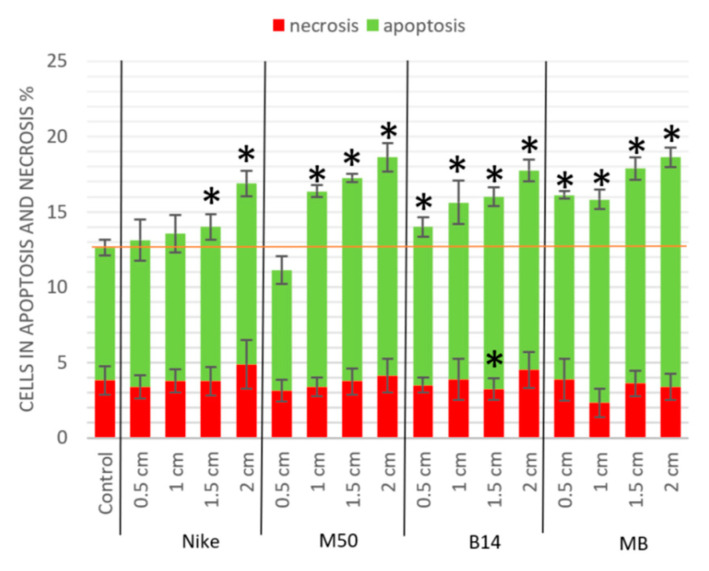
Apoptosis of the THP-1 cells after 48 h of incubation with the tested linen fabrics on four different surfaces (0.5, 1.0, 1.5 and 2.0 cm). The results are presented as the percentage of apoptotic cells. The results are the means of 5 independent experiments. The statistical significance of the differences between the results for the tested linen fabric compared to the control was calculated using Tukey’s post hoc test (* *p* < 0.05).

After 48 h of incubation of THP-1 cells with flax fabrics, the percentage of cells in necrosis increased the number of cells in apoptosis across the entire range of B14 fabric surfaces tested and the combination of M50 and B14 fabrics. The increase was dependent on the area of the test fabric (Figure 5). The lowest number of cells in the apoptotic phase was observed in cultures treated with M50 fiber in an area of 0.5 cm. As for the traditional fabric—Nike—only 1.5–2.0 cm areas caused a statistically significant growth of cells in apoptosis. Necrosis is not statistically significant. It is available only for one fabric 1.5 cm thick.

### 3.7. Cell Cycle

**Figure 6:**
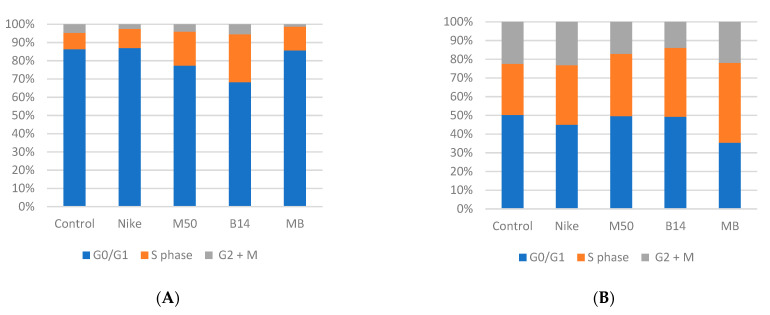

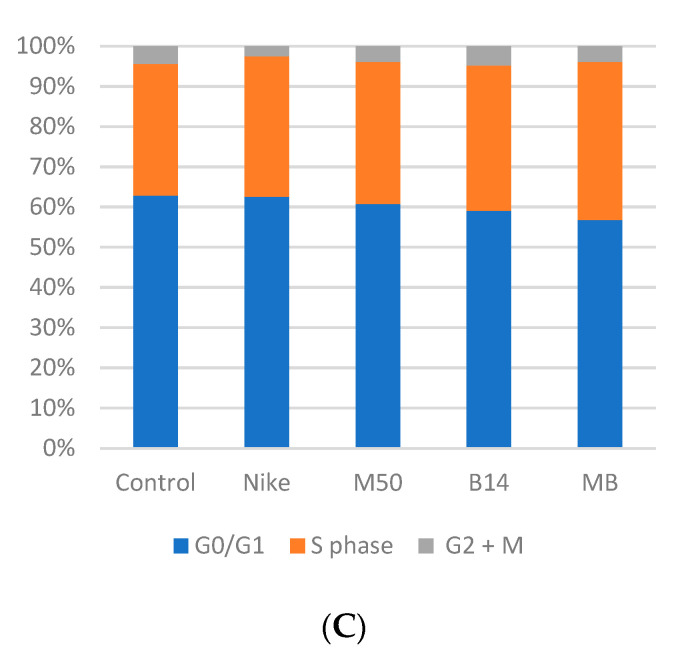
The cell cycle in HEMVEC (**A**), NHEK (**B**) and THP-1 (**C**) cells after 48 h incubation.

Incubation of the cultures with the tested linen fabrics increased the number of cells in the proliferative phase (S phase) (Figure 6). At the same time, the greatest increase was observed after using the B14 fabric.

## 4. Discussion

The introduction of a new medicinal preparation to the market is preceded by a labor-intensive process of developing not only the drug molecule itself but also its form and its approval for use in treatment based on the results of clinical trials, which means that the patient receives a proven, effective and safe product that meets his specific health needs. In this study, it was assessed whether the technological process of obtaining linen fabrics did not lead to the loss of the healing properties of linen fibers from two transgenic types of flax (M50 and B14) and the combination of these two flax fibers (M50 + B14). All experiments were conducted in direct contact in cell cultures. The results of the obtained studies are an introduction to preclinical studies on lined dressings. Research into wound-healing linen dressings is conducted by a few research centers in the world. The following briefly presented results of previous studies showing the effect of the content of active ingredients, such as antioxidants, contained in dressings on wound healing and mechanical properties.

Earlier, our research presented that the transgenic flax plants that contained elevated levels of polyphenolic compounds (ferulic acid, coumaric acid, or 4-hydroxybenzoic acid) with proven antioxidant and pro-proliferative effects) significantly improve skin wound healing. What is more, none of the linen fabrics tested were cytotoxic to the fibroblast cultures, and they did not significantly increase the incidence of cell apoptosis in the cultures. In the comet test, the linen fabrics tested showed a significant protective effect on DNA damage caused by the addition of H_2_O_2_ to the culture at the end of the incubation time. Fabrics made of B14 and MB flax are the strongest activators of NHDF cells in the in vitro tests used; hence, they can be recommended for the development of a new type of bandage capable of improving the healing of skin wounds [21]. Reactive oxygen species are responsible for the pathogenesis of chronic wounds and the prevention of wound healing as they reduce the ability to proliferate. That is why the presence of antioxidants in the composition of linen dressings violates wound-healing processes [22].

The M50 fibers produced the polyhydroxybutyrate (PHB); therefore, it had improved mechanical properties [23]. In addition, polyhydroxybutyrate (PHB) degrades in contact with body fluids, releasing monomers that activate cell proliferation [24]. What is more, M50 fibers raised the viability of NHDF, and they did not have an impact on the morphology of the cells [25].

A 12-week study of flax dressings applied to non-healing venous leg ulcers (lasting at least 2 years; 7 years on average) was carried out alone or in combination with an oil emulsion and/or seed extract. It was presented that the application of a modified linen dressing accelerated healing and reduced the exudation and size of the wound. In addition, the modified linen dressing reduced the pain associated with chronic venous ulceration [22].

The healing of chronic ulcers can be supported by antioxidant compounds [22], which have a favorable effect on wound healing, as shown in studies in animal models [25]. Linoleic acid (LA) and alpha-linolenic acid (ALA) are the most abundant in flax fibers, which may decrease the amount of arachidonic acids and other pro-inflammatory eicosanoids [26].

In another study, chronic wounds were treated with a new dressing made of genetically modified flax that contained powerful antioxidants (phenolic acids, lignans). Treatment with the new linseed dressing successfully reduced wound exudate, fibrin levels and ulcer size [22]. Ideal dressings should provide moist, semi-permeable and antiseptic environments. Moreover, the dressings should allow visual observation of the wound without having to remove them, thus reducing the number of dressings in the lesion [27]. Research has also been conducted on flax substrates modified with silver nanoparticles, which also promote wound healing [28].

Six active dressings from different categories were tested for acute irritation in rabbits and for sensitization in guinea pigs. Only a dry flax fiber dressing may have a negligible risk of causing allergic reactions in humans compared to hydrogel dressing, chitosan sponge dressing and silver nanoparticles [29].

The fabric obtained from genetically modified flax accelerates healing and contains cannabidiol, which has an analgesic effect. In addition, due to its very strong hygroscopic properties, it absorbs exudate and cleans the tissue, which is especially important in deep wounds containing large amounts of exudate. It has been found in human studies that dressings made of transgenic flax do not cause chafing of healthy skin around the dressing [22].

## 5. Conclusions

M50, B14 and MB linen fibers of modified linen have even better properties than traditional linen. In addition to this, the necrosis level was higher for conventional flax compared to the modified flax. Modified varieties of flax have a favorable effect on tissue cultures [30]; therefore, linen fabrics were made on their basis—the research on which is presented in this publication. It turned out that the technological process was correct and did not change the properties of the flax fibers. All the tested linen fabrics did not raise the number of necrotic cells compared to the control cultures. Moreover, the tested fabrics caused a statistically significant decrease in the total protein content in skin cancer. Incubation of the cultures with the tested linen fabrics caused the growth of cells in the proliferative phase for all fabrics, but the greatest increase was observed with the B14 fabric. The obtained result shows that the wound-healing properties have been preserved, so further in vivo studies of the obtained linen fabrics are possible.

## Figures and Tables

**Table 1:** Content of hydrophilic bioactive compounds per cm^2^ of fabric—the measurement was made after the sterilization to also take into account any losses arising in this process (average of 5–6 independent samples obtained from different parts of the fabric). The statistical significance of the differences between the results for the tested linen fabrics compared to the control was calculated using the Tukey post hoc test (* *p* < 0.05).

µg/cm^2^	Nike	B14	M50	MB
wanilin	2.739 ± 0.342	2.015 ± 0.333 *	2.599 ± 0.273 *	2.349 ± 0.328 *
4-hydroxy-benzoic acid	0.522 ± 0.05	0.437 ± 0.03 *	0.855 ± 0.33 *	0.621 ± 0.05
vanilic acid	1.304 ± 0.04	0.612 ± 0.03 *	1.046 ± 0.04 *	0.760 ± 0.08 *
ferulic acid	0.430 ± 0.03	0.369 ± 0.04 *	0.459 ± 0.04*	0.410 ± 0.05 *
p-coumaric acid	0.253 ± 0.06	0.118 ± 0.02 *	0.207 ± 0.04 *	0.142 ± 0.04 *
syringic aldehyde	0.391 ± 0.03	0.464 ± 0.04 *	0.519 ± 0.03 *	0.509 ± 0.05 *
syringic acid	0.209 ± 0.01	0.174 ± 0.01 *	0.277 ± 0.02 *	0.201 ± 0.04
acetosyringon	0.499 ± 0.03	0.372 ± 0.06 *	0.486 ± 0.04	0.375 ± 0.04 *
acetovanilon	0.258 ± 0.01	0.174 ± 0.02 *	0.308 ± 0.02 *	0.240 ± 0.04
orientin (luteolin-8-C-glucoside)	0.235 ± 0.03	0.121 ± 0.03 *	0.121 ± 0.015 *	0.103 ± 0.01 *
witexin (apigenin-8-C-glucoside)	0.874 ± 0.05	0.587 ± 0.07 *	0.980 ± 0.052 *	0.751 ± 0.04 *

**Table 2:** Content of other bioactive compounds per cm^2^ of fabric—the measurement was made after the sterilization (average of 5–6 independent samples obtained from different parts of the fabric). The statistical significance of the differences between the results for the tested linen fabrics compared to the control was calculated using the Tukey post hoc test (* *p* < 0.05).

µg/cm^2^	Nike	B14	M50	MB
canabidiol	0.029 ± 0.009	0.017 ± 0.003 *	0.024 ± 0.003 *	0.025 ± 0.002 *
lutein	0.015 ± 0.005	0.014 ± 0.005	0.031 ± 0.004 *	0.022 ± 0.001 *
polyhydroxybutyrate/ hydoxybutyrate	0.034 ± 0.003	0.021 ± 0.003 *	0.035 ± 0.002	0.028 ± 0.003 *
β-sitosterol	0.667 ± 0.007	0.526 ± 0.005 *	0.964 ± 0.003 *	0.753 ± 0.004 *
alfa-linolenic acid (C18:3)	2.300 ± 0.093	1.752 ± 0.003 *	2.870 ± 0.099 *	3.150 ± 0.003 *
linoleic acid (C18:2)	7.820 ± 0.056	7.592 ± 0.075	12.095 ± 0.108 *	9.275 ± 0.083 *
oleic acid (C18:1)	25.530 ± 0.255	17.520 ± 0.331 *	24.805 ± 0.333	19.425 ± 0.299 *
stearic acid (C18:0)	88.320 ± 1.003	61.612 ± 0.663 *	79.745 ± 0.655 *	69.825 ± 0.991 *
palmitic acid (C16:0)	152.260 ± 0.999	104.682 ± 0.456 *	130.175 ± 0.666 *	117.775 ± 0.566 *
unsaturated fatty acids	35.650 ± 0.450	26.864 ± 0.450 *	39.770 ± 0.550 *	31.850 ± 0.550 *
saturated fatty acids	240,580	166,294 *	209,920 *	187,600 *
Poliamines	2.369 ± 0.055	5.995 ± 0.033 *	5.232 ± 0.045 *	5.306 ± 0.056 *

## Data Availability

Data are available from Tomasz Gębarowski (Tomasz.gebarowski@upwr.edu.pl).
